# Typhoid Outbreaks, 1989–2018: Implications for Prevention and Control

**DOI:** 10.4269/ajtmh.19-0624

**Published:** 2020-03-30

**Authors:** Grace D. Appiah, Alexandria Chung, Adwoa D. Bentsi-Enchill, Sunkyung Kim, John A. Crump, Vittal Mogasale, Rachael Pellegrino, Rachel B. Slayton, Eric D. Mintz

**Affiliations:** 1 Division of Foodborne, Waterborne and Environmental Diseases, Centers for Disease Control and Prevention, Atlanta, Georgia;; 2 Usher Institute of Population Health Sciences and Informatics, University of Edinburgh, Edinburgh, United Kingdom;; 3 Department of Immunization, Vaccines and Biologicals, World Health Organization, Geneva, Switzerland;; 4 Division of Infectious Diseases and International Health, Duke University Medical Center, Durham, North Carolina;; 5 Centre for International Health, University of Otago, Dunedin, New Zealand;; 6 Policy and Economic Research Department, Development and Delivery Unit, International Vaccine Institute, Seoul, South Korea;; 7 Johns Hopkins University School of Medicine, Baltimore, Maryland;; 8 Division of Healthcare Quality and Promotion, Centers for Disease Control and Prevention, Atlanta, Georgia

## Abstract

Typhoid fever remains an important public health problem in low- and middle-income countries, with large outbreaks reported from Africa and Asia. Although the WHO recommends typhoid vaccination for control of confirmed outbreaks, there are limited data on the epidemiologic characteristics of outbreaks to inform vaccine use in outbreak settings. We conducted a literature review for typhoid outbreaks published since 1990. We found 47 publications describing 45,215 cases in outbreaks occurring in 25 countries from 1989 through 2018. Outbreak characteristics varied considerably by WHO region, with median outbreak size ranging from 12 to 1,101 cases, median duration from 23 to 140 days, and median case fatality ratio from 0% to 1%. The largest number of outbreaks occurred in WHO Southeast Asia, 13 (28%), and African regions, 12 (26%). Among 43 outbreaks reporting a mode of disease transmission, 24 (56%) were waterborne, 17 (40%) were foodborne, and two (5%) were by direct contact transmission. Among the 34 outbreaks with antimicrobial resistance data, 11 (32%) reported Typhi non-susceptible to ciprofloxacin, 16 (47%) reported multidrug-resistant (MDR) strains, and one reported extensively drug-resistant strains. Our review showed a longer median duration of outbreaks caused by MDR strains (148 days versus 34 days for susceptible strains), although this difference was not statistically significant. Control strategies focused on water, sanitation, and food safety, with vaccine use described in only six (13%) outbreaks. As typhoid conjugate vaccines become more widely used, their potential role and impact in outbreak control warrant further evaluation.

## INTRODUCTION

Typhoid fever is a systemic febrile illness caused by *Salmonella enterica* serovar Typhi (Typhi) responsible for an estimated 11–21 million illnesses and 65,000–188,000 deaths worldwide each year.^1–6^ In areas of Asia and sub-Saharan Africa, with high typhoid incidence (> 100 cases/100,000 persons per year), large outbreaks with Typhi strains resistant to multiple antimicrobials^7–11^ have been reported from both rural and urban settings where access to safe food, water, and sanitation is limited. Increasing resistance has limited antimicrobial treatment options: multidrug-resistant (MDR) strains, defined as resistant to the three former first-line antimicrobial agents (ampicillin, chloramphenicol, and trimethoprim–sulfamethoxazole), have been widespread in Asia since the early 1990s and have been increasing in many regions of Africa.^12^ Consequently, treatment with ciprofloxacin increased since the late 1990s, with the associated widespread fluoroquinolone resistance emerging among Typhi isolates from Asia and parts of Africa. In 2016, the first outbreak of an extensively drug-resistant (XDR) Typhi strain, with resistance to ceftriaxone, ciprofloxacin, and traditional first-line agents, was reported in Pakistan.^13–15^

In addition to remediation of environmental deficiencies, such as improving access to safe drinking water and sanitation, and improvements in hygiene and food safety, the WHO recommends typhoid fever vaccination as a control strategy for both endemic and epidemic diseases.^6^ Evidence on the safety, immunogenicity, efficacy, and effectiveness of Vi polysaccharide (ViPS) and Ty21a typhoid vaccines have supported their programmatic use in endemic settings since 2008. Furthermore, randomized, controlled clinical trials in Vietnam and India^16,17^ have demonstrated that typhoid conjugate vaccines (TCVs) confer durable immunological protection among infants and young children.^18^ In 2017, Typbar-TCV became the first licensed TCV to receive WHO prequalification. Cost-effectiveness models suggest that TCVs would be highly cost-effective when administered during routine immunization in low-income countries with moderate typhoid incidence (10 to < 100 cases/100,000 person-years).^19,20^ Based on this and other evidence, in 2017, the WHO Strategic Advisory Group of Experts on immunization recommended the routine use of TCVs in children aged ≥ 6 months in typhoid-endemic countries and reemphasized the role of vaccination in response to confirmed typhoid fever outbreaks.^6,21^ A WHO position article on typhoid vaccines, published in 2018, further outlines these policy recommendations.^6^ Interim analysis from a recent phase 3 clinical trial of Typbar-TCV in Nepal also showed high vaccine protective efficacy (81.6%) from a single dose among children aged 9 months to < 16 years.^22^ In addition, Gavi, the Vaccine Alliance, approved funding to support the routine introduction of TCV in typhoid-endemic countries from 2019 through 2020. In 2019, Pakistan became the first country to initiate Gavi-funded introduction of TCV into routine immunization. In view of more frequent outbreaks of drug-resistant Typhi, there is an urgent need for optimized typhoid fever control, integrating new vaccination strategies with proven water, sanitation, and hygiene (WASH), and food safety interventions.

Before these recent advances in TCV availability, implementation of the WHO recommendation for typhoid vaccination in endemic countries was rare; implementation of the recommendation for the TCV use is still in an early stage.^21^ Although typhoid vaccination is recommended for outbreak control, data that could inform decision-making on the effective use and timing of vaccination campaigns are limited; such data would include typhoid outbreak characteristics (e.g., size, duration, and cost), and the effectiveness of vaccination, WASH, or other interventions alone or in combination. Previously published reviews characterized typhoid outbreaks from mainly a local or, at most, national perspective.^23,24^ Although epidemiologic data on the global typhoid disease burden have been published,^3,5,25–30^ burden estimates focus on endemic disease and exclude data from outbreaks. Little is known about the frequency of typhoid outbreaks or their contribution to the overall disease burden, and data from outbreak investigations are sparse. We reviewed the literature for typhoid fever outbreaks published since January 1, 1990 to describe the global epidemiology of typhoid outbreaks and outbreak responses.

## MATERIALS AND METHODS

The studies included in this review were identified by independently searching three electronic databases (PubMed, Google Scholar, and Ovid), screening the reference list of an International Vaccine Institute (IVI) manuscript,^31^ and merging preexisting literature searches obtained by the U.S. CDC and the WHO. We sought to include all relevant publications on typhoid outbreaks published from January 1, 1990 through January 17, 2019. We searched databases using the following search terms: (“typhoid” or “typhoid fever” or “Salmonella Typhi” or “salmonella infection” or “enteric fever”) and (“outbreak” or “epidemic” or “burden” or “cases” or “epidemiology”). We excluded ProMED and GIDEON reports, as many of these included limited data, were unconfirmed, and reported the same outbreaks multiple times. We also excluded publications in languages other than English and those for which the full text was unavailable, duplicative, or limited to longitudinal surveillance data.

We created a merged list of publications from the databases, prior literature searches, and the IVI report, and we screened the publications for further review based on the title and abstract. Among those selected, we collected the following data: location, size, duration of outbreak, indicators of disease severity, interventions used, and antimicrobial resistance (AMR) patterns (Supplemental Table 1). We recorded the number of cases, and definitions of typhoid fever cases and outbreaks as reported by the authors, and we used WHO regional groupings to categorize outbreak locations. We defined Typhi AMR patterns as follows: susceptible strains were susceptible to all antimicrobials tested, resistant strains were resistant to at least one antimicrobial, MDR strains were resistant to all three traditional first-line agents (ampicillin, chloramphenicol, and trimethoprim–sulfamethoxazole), fluoroquinolone–non-susceptible (FQ-NS) strains were resistant or had intermediate sensitivity to ciprofloxacin, and XDR strains were MDR, FQ-NS, and resistant to ceftriaxone.

Where possible, we derived the following measures of severity and disease frequency from the abstracted data: 1) incidence proportion, or attack rate, defined as the number of new cases of disease in the population; 2) proportion of all cases with typhoid-related complications; 3) proportion hospitalized; 4) case fatality ratio (CFR), defined as the number of deaths attributed to typhoid among the total number of typhoid cases; and 5) AMR patterns. Typhoid-related complications included patients with typhoid encephalopathy, intestinal perforation, peritonitis, intestinal hemorrhage, or sepsis. Modes of transmission were categorized as waterborne, foodborne, or direct contact. For each category, we also determined the certainty of each reported source, classifying it as “suspected” if the presumed source was not tested, “probable” if the outbreak was linked by epidemiologic data to the presumed source but had samples that tested negative or were inconclusive, or “confirmed” if testing of one or more clinical samples was positive for Typhi or testing of environmental samples was positive for fecal contamination or Typhi.

Data analyses were conducted using SAS v9.4 (Cary, NC) software. The same outbreak reported in multiple publications was counted as a single outbreak; only the article with the highest case count and the most recent end date (i.e., the date of the last reported case) was used for analysis. We used the Wilcoxon signed-rank test to compare the duration of outbreaks with susceptible strains versus those with resistant strains. To better understand typhoid outbreak responses, we also supplemented the data obtained from full-text publications with a review of unpublished reports of typhoid outbreak responses obtained from direct communication with CDC and Medecins Sans Frontières (MSF).

## RESULTS

### Characteristics of included studies.

**Figure 1.**
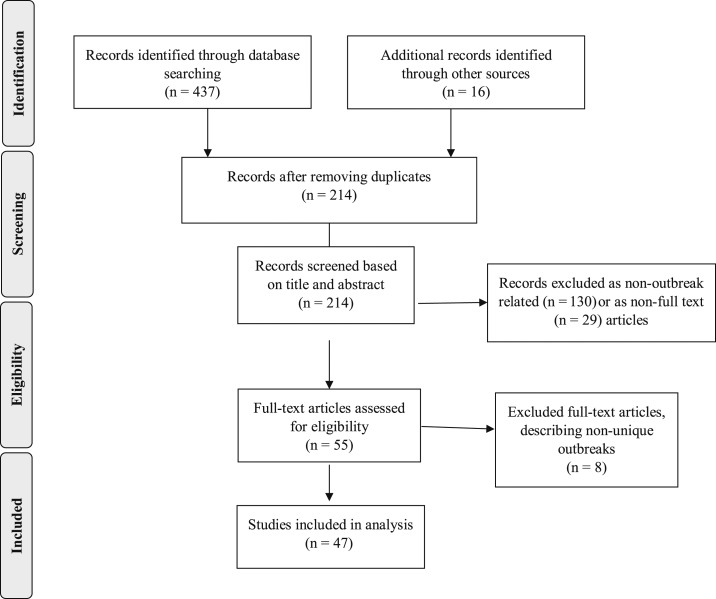
Flow diagram for literature search and selection of publications, 1989–2019.

A total of 453 publications were identified from the reference list and electronic databases (Figure 1). We excluded duplicate (239), non–outbreak-focused (130), and non–full-text (29) publications based on screening of title and abstract. Among the 55 remaining publications, we excluded eight publications describing nonunique outbreaks: Anatolia, Turkey, 2008^32,33^; Kasese, Uganda, 2009^34^; Harare, Zimbabwe, 2011^35–37^; Malawi–Mozambique border, 2012^38^; and Kampala, Uganda, 2015. One publication also described two separate outbreaks.^39^ Therefore, a total of 47 publications describing 48 unique typhoid outbreaks from 1989 to 2018 were included in the review (Table 1).

**Table 1:** Characteristics of included publications (*n* = 47)

WHO region, ref no.	Outbreak year(s)	Location	Incidence proportion	Number of cases	Number (%) hospitalized	Number (%) of complications	Number of deaths (case fatality ratio)
African							
83	2004–2005	Kinshasa, DRC	–	144	144	41 (28) IPs	64 (44)
52	2007–2009	Kasese, Uganda	–	577	289 (57)	249 (43) IPs	47 (9)
38	2009	Malawi–Mozambique	–	303	81 (27)	40 (13)	11 (4)
34	2009–2012	Kasese, Uganda	0.1%	1,341	–	568 (6)	–
50	2010–2012	Lusaka, Zambia	–	2,040	–	–	–
84	2011–2012	Kikwit, DRC	0.6%	1,430	–	71 (5)	17 (1.5)
85	2011–2012	Harare, Zimbabwe	–	3,795	–	–	–
53	2014–2015	Moyale, Kenya	–	317	–	–	2 (0.05)
49	2015	Kampala, Uganda	0.9%	10,230	–	–	–
86	2016	Tigray, Ethiopia	–	98	–	–	1
57	2016–2017	Harare, Zimbabwe	–	860	–	–	4 (0.5)
59	2017–2018	Harare, Zimbabwe	–	3,378	–	–	–
Eastern Mediterranean							
60	1992	Tabuk City, Saudi Arabia	0.1%	185	–	–	–
87	1992	Al-Mudhnab, Saudi Arabia	–	27	–	0	0
88	2004	Jordan	–	83	–	–	0
51	2004	Karachi, Pakistan	60%	300	–	–	3 (1)
15	2016–2017	Hyderabad, Pakistan	–	486	98/200 (50)	71/200 (27)	1 (0.9)
European							
47	1996–1997	Dushanbe, Tajikistan	–	10,677	–	–	108 (1)
89	1997	Utelle, France	–	26	26	–	0
90	1998	Paris, France	18%	27	21 (78)	2 (7)	0
91	2004	Leipzig, Germany	–	6	3 (50)	–	0
33	2008	Eastern Anatolia, Turkey	18.5%	867	154 (18)	8 (5)	0
Region of the Americas							
80	1989	New York	7.5%	68	21 (30)	2 (2.86)	0
66	1998–1999	Florida	–	17	14 (87.5)	–	0
45	2000	New York City	–	7	4		0
56	2000	Ohio, Kentucky, Indiana	–	9	–	–	–
67	2010	Nevada	–	12	9/11 (82%)	–	0
92	2015	Colorado	–	3	2	–	0
63	2015	Oklahoma	–	38	14 (37)	0	0
Southeast Asia							
44	1989–1990	Calcutta, India	–	117	117	–	0
93	1990	Bangalore, India	–	15	15	2	0
64	1995	Maharashtra, India	13.7%	415	–	–	0
42	1999	Thai–Myanmar border	–	11	–	2 (9)	1 (9)
61	2000	Madaya, Myanmar	2.9%	49	31 (63)	1 (3)	0
41	2002	Bharatpur, Nepal	6.5%	5,963	–	3 (0.05)	4 (0.07)
94	2007	Rajasthan, India	10.4%	219	–	–	–
62	2007	West Bengal, India	0.7%	103	6	–	0
46	2009–2010	Chandigarh, North India	–	27	–	–	–
39	2009–2010	Songkhla, Thailand	–	137	250 (70)	49 (13)	0
	2010–2011			231			
95	2014	Assam, India	2.0%	79	–	0	0
54	2015–2016	Bengaluru, India	–	24	–	–	–
Western Pacific							
48	1990	Singapore	4.8%	95	3	4	0
96	1998–1999	Nauru	–	50	32 (64)	–	0
43	1999	Xing-An, China	1.0%	24	14 (58)	–	0
58	2009	Selangor, Malaysia	–	45	–	–	–
65	2010	Shache, China	–	253	–	–	–
40	2014	Japan	–	7	–	2 (29)	0

DRC = Democratic Republic of Congo; IP = intestinal perforation.

Among the 47 included publications, all (100%) reported outbreak location, duration, and total number of cases; all but one publication reported the number of laboratory-confirmed cases. Among the confirmed cases with culture type specified, diagnosis was made by blood culture alone (8)^15,40–46^ or mixed culture types (blood, stool, and/or urine) (10). Definitions for suspected and confirmed cases were reported in only 26 (55%) and 31 (66%) publications, respectively (Supplemental Table 2); none of the publications provided a definition of a typhoid outbreak. Of the 26 case definitions for suspected cases, 25 (96%) included fever, of which 10 (40%) specified a fever of ≥ 3 days. Temperature thresholds for fever varied from 37.5°C to 38.5°C. Clinical symptoms or signs were included in 20 (77%) suspected case definitions, most commonly gastrointestinal symptoms 17 (85%). A spatiotemporal requirement, with specified outbreak dates and locations, was included in 15 (75%). The remaining seven suspected case definitions instead required clinical suspicion (2),^41,47,48^ suggestive diagnostic testing (2), or that no other cause was identified (2). Of the 12 probable case definitions, 11 (92%) included fever, of which seven (64%) specified a fever of ≥ 3 days. Clinical symptoms or signs were required in four (33%) probable case definitions; all included gastrointestinal symptoms. Of the 31 confirmed case definitions, 30 (97%) required Typhi isolation from a positive culture (stool, blood, or urine). In one definition, Widal testing was the sole criterion for case confirmation. Markers of disease severity were not always reported; 19 (40%) of 47 publications reported complications, 24 (51%) reported hospitalizations, and 35 (74%) reported deaths. Antimicrobial resistance data were reported in 34 (72%) publications.

We reviewed 14 unpublished CDC field investigation reports and one MSF epicenter report describing outbreaks in nine countries from 1986 through 2018. Among the 15 outbreaks, 11 (73%) occurred in low- and middle-income countries (LMICs) and four (27%) in the United States. Three (20%) outbreaks (Marshall Islands, 1986, 2006; Thailand, 1999) were not previously captured in the published literature.

### Characteristics of outbreaks.

Forty-eight typhoid outbreaks occurred in 25 countries and accounted for a total of 45,215 cases: 13 (27%) occurred in WHO Southeast Asia region, 12 (25%) in the African region, seven (15%) in the region of the Americas, six (13%) in the Western Pacific, and five (10%) each in the Eastern Mediterranean and European regions (Table 1). The median number (range) of cases reported was highest in the African region, 1,101 (98–10,230), followed by the European region, 332 (6–10,677). The highest case counts in these regions were reported from prolonged outbreaks in Uganda^49^ (10,230 cases in 539 days) and Tajikistan^47^ (10,677 cases in 551 days). Overall, 5,777 (13%) of 45,215 cases were culture confirmed, at least 314 (5%) of which were known to be confirmed by blood culture.

The median (range) outbreak duration was 57 (6–989) days, with the longest duration in the African region, 140 (13–989) days, followed by the Western Pacific region, 101 (15–219) days. The longest reported outbreak (989 days) was in Zambia.^50^ The median incidence proportion (range), 5% (0–60%), was highest in the Eastern Mediterranean region because of an outbreak in Pakistan,^51^ in which 300 (60%) cases were reported among 500 village residents with exposure to the same fecally contaminated well water. Complications including bowel perforation, gastrointestinal bleeding, and meningitis were reported in 15 outbreaks (Table 1). The single outbreak with the highest proportion of complications reported was in Kasese district of Uganda^52^; 249 (43%) of 577 suspected cases had intestinal perforation with peritonitis.

The median (range) proportion hospitalized was 50% (3–82%): highest in the Southeast Asia region, 63% (6–71%), and lowest in the Eastern Mediterranean region (20%). The median (range) CFR was 0% (0–44%), with the highest CFR in the African region, 1% (0.1–44%), due in large part to an outbreak in Kinshasa, Democratic Republic of Congo (DRC), in which 64 (44%) deaths were reported among a subset of 144 patients undergoing surgery for peritonitis. The overall CFR during the outbreak in the DRC, 1% (134 deaths among 13,400 suspected cases), was consistent with the average for the region.

### Antimicrobial resistance patterns.

Among 34 outbreaks with Typhi AMR data, 11 (32%) were FQ-NS, 16 (47%) were MDR, one was XDR, and 25 (74%) had isolated resistance to at least one antimicrobial (Table 2). Ceftriaxone resistance was reported in three outbreaks,^15,53,54^ including the XDR typhoid outbreak in Pakistan. No outbreak strains were reported as resistant to azithromycin. The median duration of 16 outbreaks caused by MDR Typhi was 148 days, compared with a median duration of 34 days among 10 outbreaks caused by pan-susceptible strains (*P* > 0.05). The median duration for the 11 outbreaks with FQ-NS strains and the three with ceftriaxone-resistant strains was 128 days and 119 days, respectively.

**Table 2:** Characteristics of typhoid outbreaks (*n* = 48), by WHO geographic region, 1989–2018

Characteristic	WHO region*						Total
	African	Eastern Mediterranean	European	Region of the Americas	Southeast Asia	Western Pacific	
Outbreak years	2004–2018	1992–2017	1996–2008	1989–2015	1989–2016	1990–2014	1989–2018
Number of countries reporting outbreaks	8	3	4	1	4	5	25
Number of outbreaks	12	5	5	7	13	6	48
Number multidrug resistant	6	3	2	0	4	1	16
Number fluoroquinolone non-susceptible	6	1	0	0	2	2	11
Total number of cases	24,513	1,081	11,603	154	7,390	474	45,215
Median cases (range)	1,101 (98–10,230)	185 (27–486)	332 (6–10,677)	12 (3–68)	91 (11–5,963)	48 (7–253)	101 (3–10,677)
Number of confirmed cases	848	630	3,370	110	607	212	5,777
Median duration days (range)	140 (13–989)	60 (11–390)	23 (6–539)	50 (23–139)	36 (11–304)	101 (15–219)	57 (6–989)
Median incidence proportion (range)	0.2% (0–0.3%)	60% (60–60%)	16% (14–40%)	8% (8–8%)	3% (0.7–14%)	3% (1–5%)	5% (0–60%)
Median proportion hospitalized (range)†	27% (17–50%)	20% (20–20%)	34% (12–78%)	62% (31–82%)	63% (6–71%)	58% (3–64%)	50% (3–82%)
Median case fatality ratio (range)	1% (0.1–44%)	0% (0–1%)	0% (0–1%)	0% (0–4%)	0% (0–9%)	0% (0–0%)	0% (0–44%)

CFR indicates proportion of all cases that died.

* Twenty-five countries [number of outbreaks] reviewed by WHO region: African (Uganda [3], Zimbabwe [3], Democratic Republic of Congo [2], Ethiopia, Kenya, Malawi–Mozambique border, and Zambia), Eastern Mediterranean (Pakistan [2], Saudi Arabia [2], and Jordan), European (France [2], Turkey, Tajikistan, and Germany), region of the Americas (the United States [7]), Southeast Asia (India [8], Thailand [2], Thai–Myanmar border, Myanmar, and Nepal), and Western Pacific (China [2], Japan, Malaysia, Nauru, and Singapore).

† Incidence proportion, or attack rate, is the number of ill persons in the exposed population.

### Modes of transmission.

**Figure 2.**
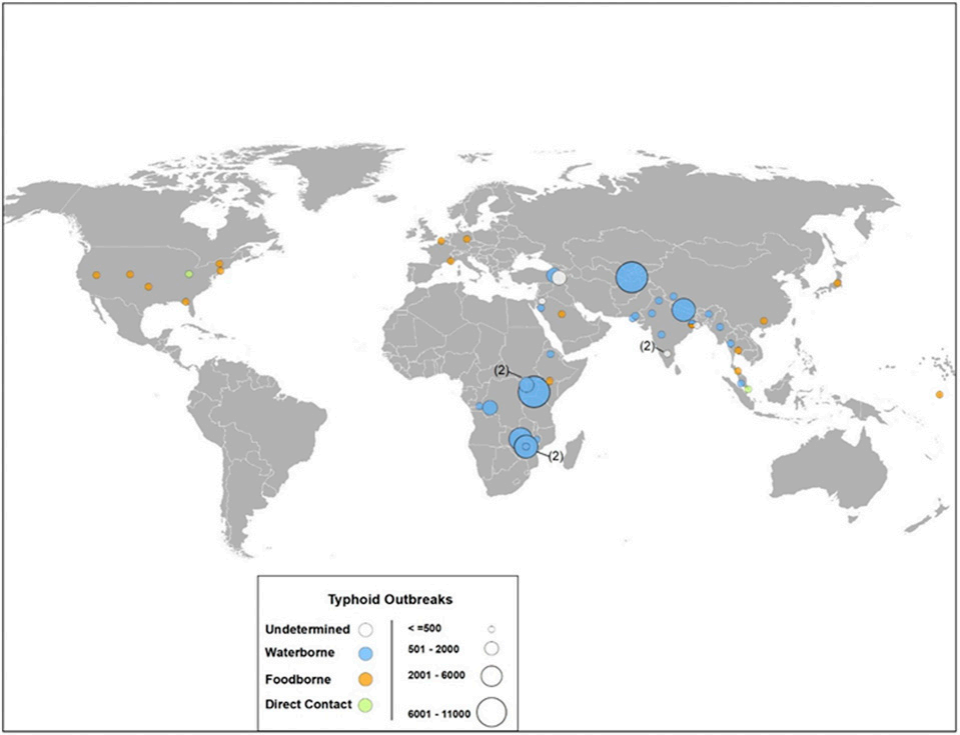
Distribution of typhoid outbreaks by country (*n* = 43), size, and mode of transmission, 1989–2018. This figure appears in color at www.ajtmh.org.

Forty-three outbreaks reported a mode of transmission: waterborne 24 (56%), foodborne 17 (40%), and direct contact 2 (5%) (Figure 2). The average number of cases per outbreak was largest for waterborne outbreaks (1,660), as compared with that for outbreaks with foodborne (58) or direct contact transmission (52). The source of water contamination was found in 18 (42%) outbreaks and included burst pipes, low-pressure water distribution systems, lack of chlorination, and drinking water sources in close proximity to latrines (Supplemental Table 3). For example, an environmental water quality survey during the 2015 typhoid outbreak in Kampala, Uganda, found unregulated drinking water sources to be a mode of typhoid transmission.^55^ A food vehicle was associated with six (14%) outbreaks, in which the outbreak was linked to a food handler and/or asymptomatic carrier. Two outbreaks, one with sexual transmission in men who have sex with men^56^ and one in an inpatient psychiatric ward, were reported as direct contact transmission.

### Interventions.

Among 48 published outbreaks, 47 (98%) mentioned interventions and 26 (54%) provided details about their implementation. Although multiple intervention strategies were discussed, the types of interventions implemented generally were classified into five broad categories: 1) improving WASH infrastructure, 2) other measures to improve drinking water quality, 3) increasing health education, 4) revising policy and treatment guidelines, and 5) typhoid vaccination. The most commonly implemented intervention, described in 17 (65%) of 26 outbreaks, was improving WASH infrastructure, including building a new water tower, repair of unsanitary latrines and toilets, and rehabilitation of fecally contaminated boreholes.^38,51,57–59^ Other measures to improve drinking water, implemented in 14 (54%) outbreaks,^32,38,39,44,47,49,57,59–65^ were described as improving water quality which included providing alternative drinking water sources and distribution of chlorine products for water disinfection in the household, point of use, point of water collection, or at the municipal water source. Efforts to increase health education were described in 15 (58%) outbreaks. Policy revisions and treatment recommendations were described in five (19%) outbreaks: revising food preparation guidelines, closing underground water sources,^49^ product recalls,^66,67^ and in one outbreak, the treatment of a food handler who was a suspected convalescent carrier of Typhi.

Vaccine use was described in six outbreaks: ViPS vaccine in 5^43,48,64,65,68^ and TCV in one outbreak. In two community typhoid outbreaks, the use of vaccines during the outbreak was only briefly mentioned. In a 2010 outbreak of MDR Typhi, in which 198 suspected and 55 laboratory-confirmed cases were detected, administering the ViPS vaccine was noted among other interventions (improving WASH and increasing health education) credited with ending the outbreak within 5 months. However, no details were provided on the timing or effectiveness of vaccination. In a 1995 typhoid outbreak in India that was linked to well water use, 86 suspected and 22 laboratory-confirmed cases were reported from one village. Chemoprophylaxis and vaccination of contacts were listed as prevention measures, but no additional details on the type of vaccine, implementation, or effectiveness were provided. In 2010, after a category four cyclone in Fiji, a preemptive ViPS vaccination campaign, in children and adults aged ≥ 2 years, was conducted in cyclone-affected areas, where typhoid was considered to be endemic. Vaccine was also administered in a few areas that were not affected by the cyclone but where typhoid outbreaks had recently occurred. An impact evaluation showed a reduced annual typhoid fever incidence during the postvaccination year compared with previous years in vaccinated areas, but increased or no change in typhoid incidence in unvaccinated areas.^68,69^

Two reactive ViPS vaccination campaigns were conducted in institutional settings; in 1990, during an outbreak in a psychiatric hospital in Singapore,^48^ and in 1999, during an outbreak at a secondary school in China.^43,69^ In the Singapore outbreak, the suspected transmission was through close person-to-person contact; patients and staff were given two doses of the ViPS vaccine within 2 months of the outbreak onset. That outbreak ended 3 months later, with control attributed to improved environmental sanitation, active case finding, screening of asymptomatic persons, and the vaccination campaign. Vaccine efficacy was estimated to be 66% after the second dose, with no protection conferred after the first dose. In southwestern China, a reactive vaccination campaign with ViPS was initiated within 1 month of an outbreak in a middle school. Of note, many children attending the school were previously vaccinated during routine ViPS typhoid vaccination the prior year. The outbreak ended 1 month after the 3-day vaccination campaign ended. Among the 2,111 total students, 1,701 were vaccinated. An analysis of vaccine effectiveness determined that ViPS was associated with 71% protection against typhoid fever, supporting the use of typhoid vaccine for outbreak control.

In response to the first reported XDR typhoid outbreak, which began in Hyderabad district of Sindh Province, Pakistan, in October 2016, the first reactive TCV campaign was launched in March 2018. The attack rate was highest in children aged ≤ 15 years; the campaign targeted children aged 6 months to 10 years,^70^ administering 250,000 doses in Sindh Province.^71^ A total of 10,365 confirmed XDR cases had been reported in Sindh Province as of August 25, 2019.^72^ An expanded TCV campaign, targeting approximately 10 million children aged 9 months to < 15 years, was conducted in Sindh Province in November 2019.^73^ An evaluation of the impact of vaccination is planned in 2020.

In the prolonged typhoid outbreak in Kasese, Uganda, in 2009, vaccines were not used, but a later modeling study showed that emergency vaccination would have been a cost-effective control measure.^74^ Qualitative interviews conducted among local communities during a 2009–2010 typhoid outbreak in Malawi showed high sociocultural acceptance of vaccination for typhoid control.^75^ By contrast, during the same outbreak, perceptions of WASH effectiveness and the use of a household-level water treatment intervention were low, despite educational messaging.^76^

During the large-scale, prolonged typhoid outbreak in Dushanbe, Tajikistan, the emergence of MDR and ciprofloxacin-resistant Typhi later prompted review of global policies regarding mass typhoid vaccination and recommendation for its use as an adjunct to WASH interventions, for rapid outbreak control.^77^ The reviewers suggested the following potential indications for vaccination during global typhoid outbreaks: sustained, high-incidence outbreaks; waterborne outbreaks with evidence of multiple point sources of water contamination; outbreaks with drug-resistant Typhi strains; and settings in humanitarian crisis without immediate resources to implement effective water and sewage infrastructure improvements.

## INTERVENTIONS IN UNPUBLISHED REPORTS

In the 14 unpublished CDC outbreak reports, at least one of four categories of response activities was described in all outbreaks: 1) epidemiologic investigations, 2) environmental testing of water sources to identify fecally contaminated and/or inadequately chlorinated water, 3) WASH improvements, and 4) health promotion activities. Investigational studies were performed in all 14 reported outbreaks and included case–control studies to assess transmission risk factors, hospital-based case series, household investigations, community assessment surveys for WASH measures, and studies to examine the incidence of complications such as intestinal perforation and neurological sequelae.

Environmental testing of public or household-level drinking water sources for *Escherichia coli* and/or free chlorine residuals was described in nine (90%) of 10 outbreaks in LMICs. WASH improvements were heterogeneous, with interventions in the nine outbreaks ranging from distribution of household-level water purification tablets, provision of alternate drinking water sources to large-scale repair of broken boreholes, placement of in-line chlorinators, and closing of non-treated groundwater sources. Health promotion activities included increasing community awareness of both typhoid fever symptoms and prevention strategies, and improving provider education on typhoid case management. In the four suspected foodborne outbreaks in the United States, educational messaging on hygienic food preparation for food handlers and food safety inspections were carried out.

The MSF Epicentre^43^ report also described a reactive ViPS vaccination campaign in 1999 following an outbreak of 185 typhoid cases in a Myanmar refugee camp in Thailand. The vaccination campaign, initiated 2 months after the outbreak onset, targeted 12,593 children and adolescents with an estimated 75–88% vaccine efficacy.^78^

## DISCUSSION

We identified 48 typhoid fever outbreaks in the published literature occurring from 1989 through 2018 and three additional outbreaks from unpublished reports. In accordance with estimates of typhoid fever burden,^25–28^ most outbreaks occurred in Southeast Asia and sub-Saharan Africa. Although in a predictive model Oceania was the subregion expected to have the highest typhoid fever incidence per capita (based on having the lowest coverage of improved drinking water and improved sanitation),^2^ we found only one reported outbreak from the region.

Outbreaks in the WHO African region had the longest median duration and the highest CFR. Fecally contaminated water was the most commonly identified outbreak source, consistent with data from informal settlements linking poor water and sanitation infrastructure to increased typhoid transmission.^19,41,79^ High disease severity markers in two outbreaks in the African region were likely due to selection bias. In the DRC, high CFRs were reported among a subset of surgical patients with peritonitis. In Kasese, Uganda, high rates of intestinal perforation were detected after conducting case finding among hospitalized patients diagnosed with intestinal perforation.

In high-income countries with improved drinking water sources, food safety, and sanitation infrastructure, outbreaks were more often foodborne, culture confirmed, and of relatively short duration.^23,66,67,80,81^ In general, the data in this review showed that waterborne typhoid outbreaks in LMICs were large and prolonged. Over time, outbreaks globally were increasingly associated with drug-resistant Typhi strains. Our review showed a longer median duration of outbreaks caused by drug-resistant strains, although this difference did not reach statistical significance.

Data were insufficient to determine the impact of vaccination alone or in combination with other interventions to curtail the spread of typhoid in outbreak settings. However, these data could provide useful parameters for mathematical modeling studies; improving identification of outbreaks where reactive vaccination may be an effective intervention. In the few reactive vaccination campaigns reviewed (five with ViPS vaccine and one with TCV), vaccination was reported to be effective, when paired with WASH interventions and education, in curtailing outbreaks.^77,82^ The few qualitative studies of perceptions of typhoid vaccination during outbreaks suggest high vaccine acceptance.^75,76^

Our review is subject to several limitations. First, we relied on the published literature, which may not represent true global patterns in outbreaks because of data deficiencies (including lack of data from many LMICs), heterogeneity in outbreak detection capacity, reporting biases, and population size by location. Case counts were not adjusted by regional population size, and population-level data were lacking to compare incidence or attack rates, thereby making interregional comparisons less accurate.

Second, we accepted the authors’ use of the term “outbreak” and reported case definitions which may have been overly inclusive, as we did not have sufficient primary data in all articles to apply a standard definition. Comparisons between heterogeneous outbreaks with different case definitions and laboratory methods ranging from culture confirmation to the use of serologic tests limited the specificity of our findings.

Third, few publications provided sufficient details on the timing and effectiveness of outbreak interventions to determine which activities had the greatest impact on outbreak control. Although the unpublished CDC outbreak reports were more informative, none of those reports described vaccine use in the respective outbreak. Finally, with the exception of the 2018 TCV campaign in Sindh Province, Pakistan, the few reactive vaccination campaigns described in detail in our review were limited to institutional or post-disaster settings, and these findings may not be more broadly applicable to outbreaks in wider community settings.

Despite the limitations, our findings provide valuable data on the global epidemiology of typhoid outbreaks and highlight the need for standardization in outbreak detection and monitoring. For example, although outbreak settings are heterogenous, standardized case definitions should be used to identify patients for culture confirmation of suspected typhoid, standardized reporting definitions should be applied to distinguish true outbreaks from variations in endemic typhoid fever, and increased outbreak detection and reporting should be prioritized from regions, including Oceania, where current data are lacking. Vaccine effectiveness data from future reactive vaccination campaigns, including robust data on the timing and impact of vaccine use, and mathematical modeling studies of transmission patterns and immunization strategies, are needed to more accurately inform decision-making on vaccine use during outbreaks.

## Supplemental tables

Supplemental materials
