# Supplementary material for: Typhoid Outbreaks, 1989–2018: Implications for Prevention and Control
Source: Am J Trop Med Hyg. 2020 Mar 30;102(6):1296–305. doi: 10.4269/ajtmh.19-0624 (PMC7253085; doi:10.4269/ajtmh.19-0624)
Supplement: Supplementary file 1 [file tpmd190624.SD1.docx]

The following are supplemental materials and will be published online only

**Supplemental Appendix**

Title: Typhoid Outbreaks, 1989-2018: Implications for Prevention and Control

**Supplemental Material:**

**List of Contents:**

Supplemental Table 1. List of Data Extraction Terms

Supplemental Table 2. Case Definitions

Supplemental Table 3. Modes of Transmission and Sources

**Supplemental Table 1. List of Data Extraction Terms**

| **Section** | **Data collected** |
| --- | --- |
| *Data from Studies^1^* | lead author, publication year, location, WHO region, type of study, reference |
| Cases and Duration | outbreak definition, case definitions (suspected, probable, confirmed), number of cases (suspected, probable, confirmed and all), notes on definitions, if depends on clinical diagnosis, confirmed (all, blood, non-blood), comments on culture confirmation, outbreak dates (peak date, first case, last case, decade of outbreak), and duration |
| Measures of Disease Frequency | Incidence proportion, proportion hospitalized, proportion with complications, case fatality rate |
| Interventions | Identification of sources (suspected, probable or confirmed), type of investigation carried out, response interventions considered, interventions carried out (vaccine use, WASH, water source cleaning, removal of food handlers, replacement of water source, policy/guideline change), |
| AMR Patterns | AMR patterns (MDR, XDR, FQ-NS), antimicrobials used for treatment |
| *Data about Studies^2^* | percentage of studies reporting: location, number of cases, outbreak definition, case definitions (suspected, probable, confirmed), laboratory confirmation (total and blood), duration, age (ranges, median), dates of outbreak, count of investigations reporting suspected sources, measures of disease frequency, interventions considered and carried out, vaccine use and antimicrobial testing |
| ^1^Data from studies were collected in Excel spreadsheets; ^2^Data about studies were compiled in Excel pivot tables | |

**Supplemental Table 2. Reported Typhoid Fever Typhoid Fever Case Definitions (*n*=37)**

| **Ref No.** | **Suspected** | **Confirmed** |
| --- | --- | --- |
| 84 | "Fever ≥ 38°C for more than three days and digestive disorders (diarrhea, constipation, abdominal pain) and has a negative malaria test" | "Suspected case confirmed by isolation of S. Typhi from blood, bone marrow or duodenal fluid" |
| 60 | "Any patient during the outbreak period (April-June 1992) for whom the differential diagnosis included typhoid fever and for which no other confirmed diagnosis was established" | "A suspect case of TF with *Salmonella typhi* isolated from blood, stool, urine or other clinical specimen" |
| 87 | “A case of fever and other symptoms suggestive of typhoid fever with a high Widal test for *Salmonella* Typhi O antigen but negative blood, stool or urine cultures during the period between June 1 and July 30, 1992.” | “A suspected case with Salmonella typhi isolated from blood, stool or urine” |
| 94 | "Occurrence of sustained high grade fever with or without headache, stomachache, diarrhea, and/or, constipation, vomiting, loss of appetite with negative test result for malaria in residents of Varkana village on any day since May 7, 2007" |  |
| 58 | "With fever and abdominal symptoms were identified from medical records" | "A person with or without clinical features of typhoid infection (fever and abdominal symptoms), with isolation of S. Typhi in blood or urine or stool and had visited SCRP from 26th to 27th January 2009 prior to illness onset" |
| 61 | "Person suffering from sustained fever (7 days and more) with one of the abdominal symptoms (constipation, abdominal pain, diarrhea, melena) in Mweponkan village between 1st August 2000 and 30th September 2000."  **Probable Case:** "A suspected case with positive Diazo Urine Test or positive widal test" | "Clinical symptoms with positive culture for S. typhi" |
| 33 | "A stepladder pattern of fever, headache, malaise with loss of appetite, usually with gastrointestinal symptoms and having two or more of the following symptoms/signs: toxic look; relative bradycardia; rose spots on the trunk; splenic enlargement; or non-productive cough."  **Probable Case:** "A patient with a fever lasting for at least three days 8with a positive serodiagnosis but without S. typhi isolation" | "A suspected/probable case that was confirmed by positive stool or blood culture for S. typhi" |
| 80 | **Probable Case:**  "Illness with fever of >= 38.3 C for >= 3 days with no other cause for fever in a person who had contact with hotel A during the same period" [11-14 June] | "Isolation of S. typhi from stool or blood from any person with compatible symptoms and contact with hotel A in the period 11-14 June. " |
| 62 | "Fever (temperature >38°C) for one week or more in a resident of ward-1, South Dumdum municipality […] between February and May 2007"  **Probable Case:** "A suspected case with a Widal titre of >= 1:80" |  |
| 63 | **Probable Case:** "A person with clinically compatible illness (e.g. diarrhoea, fever, etc.) who had close, personal contact to a confirmed case" | “A person with isolation of S. Typhi from a stool or blood culture, with one of the designated outbreak patterns (XbaI restriction enzyme patterns JPPX01.0255, JPPX01.1200, JPPX01.1201 or JPPX01.1029) as determined by pulsed-field gel electrophoresis (PFGE), with illness onset from 1 January through 31 March 2015” |
| 57 | "Illness that began on or after October 6, 2016, with fever ≥100.4°F (38°C), body pains, headache, and abdominal pain" in a resident of Harare City | "Blood or stool specimens positive for Salmonella Typhi" |
| 51 | "Temperature >38°C since the onset of outbreak, abdominal discomfort, diarrhea, vomiting, and weakness" | NA |
| 53 | “Any patient presenting with clinical signs and symptoms consistent with typhoid fever, including headache, fever, stomach pains, diarrhea, or chronic constipation” | “Rapid diagnostic tests (Widal), confirmatory culture from stool” |
| 92 | NA | “Clinically compatible illness with isolation of S. Typhi during July 1–October 15 and identification of an isolate with one of two pulsed-field gel electrophoresis (PFGE) outbreak patterns that differed by one band” |
| 49 | "Fever (≥37.5°C) for ≥3 days from 1 January 2015 onwards, with headache, abdominal pain, a negative test for malaria or failure to respond to anti-malaria treatment, >=2 of the following symptoms: diarrhea, nausea or vomiting, constipation, or fatigue"  **Probable Case:** "Suspected case whose serum sample yielded a positive TUBEX TF test." | “Suspected case whose blood culture yielded *S.* Typhi” |
| 66 | **Probable Case:** “Febrile illness in any Florida resident with onset during the same period and a stool, blood or urine culture from which any other laboratory identified *Salmonella* Typhi” | “Febrile illness with onset after 31 October 1998 in any Florida resident with a stool, blood or urine culture from which the state central laboratory identified *Salmonella* Typhi” |
| 64 | "Persons who had fever for 3 or more days and whose peripheral blood smear was negative for malarial parasites" |  |
| 41 | “Fever known or clinically suspected to be caused by *S.*  Enterica serotype Typhi as recorded by the admitting physician” | Suspected case who's blood culture yielded *Salmonella* Typhi [not explicitly given, but implied] |
| 67 | NA | "Illness with onset from 1 January 2010 to 30 September 2010, in a person whose specimen yielded Salmonella serotype Typhi 20matching the outbreak strain. Cases were identified through PulseNet" |
| 38 | "Illness in a resident of Neno District, Malawi, or Tsangano District, Mozambique, with onset on or after 1 March 2009, who presented with fever (subjective or with a documented temperature ≥38 C ), and at least 1 other of the following signs and symptoms: gastrointestinal illness (abdominal pain, vomiting, diarrhea, or constipation), joint pain, muscle pain, headache, neck pain or stiffness, difficulty walking or talking, arms held in flexion, or mental status changes, with no alternative explanation for the illness."  **Probable Case:** "Fever and a positive rapid typhoid test (TOBEX TF)" | "Either a blood culture yielding Salmonella Typhi, or a fever and a stool culture yielding Salmonella Typhi" |
| 86 | “Any person with gradual onset of remittent fever in the first week, headache, arthralgia, anorexia, constipation and or abdominal pain” | "A suspected case with Widal test, "O" titer of 1/160 and more is very suggestive and done for this investigation" |
| 47 | NA | "Symptoms and signs consistent with typhoid fever in a Dushanbe resident with Salmonella Typhi infection confirmed by blood or stool culture." |
| 91 | "Unexplained fever of at least 38.5°C for three or more days since 1 May 2004 and up to 31 July 2004."  **Probable Case:** "Additional positive serological reactions (Widal test and/or specific ELISA" | "Isolation of S. Typhi from blood or stool cultures" |
| 83 | NA | “Case of peritonitis for which laboratory confirmation was obtained by culture.” |
| 59 | “Fever and at least one of the following symptoms: headache, malaise, abdominal discomfort, vomiting, diarrhea, cough, or constipation.” | “Typhi isolated from blood, stool, or rectal swab culture” |
| 52 | "Illness with onset between December 27. 2007 and July 30, 2009 in a person with fever, abdominal pain, and >=1 of the following symptoms: gastrointestinal complaints (i.e. vomiting, diarrhea, or constipation), general body weakness, joint pain, headache, no response to antimalarial medication or IP" | NA |
| 96 | "Fever for ≥3 days since 1 October 1998, and one or more of the following symptoms: chills, sweats, headaches, loss of appetite, malaise, weakness, diarrhea, constipation, abdominal pain, cough, or vomiting in a resident of Nauru." | "Stool, blood or urine sample yielding S.Typhi since 1 October 1998, in a resident of Nauru with illness meeting the clinical case definition" |
| 85 | “Any person with gradual onset of steadily increasing and then persistently high fever, chills, malaise, headache, sore throat, cough, and, sometimes, abdominal pain and constipation or diarrhea” | “A suspected case confirmed by isolation of S. Typhi from blood, bone marrow or bowel fluid or stool.” |
| 89 | **Probable Case:** "An illness with fever of unknown origin (13875 C) of ^3 days’ duration occurring in a person who had stayed in Utelle between 14 and 16 August 1997" | "Isolation of *Salmonella enterica* serotype typhi from stool or blood and/or a positive Widal test from any person with compatible symptoms who had stayed in Utelle, France, between 14 and 18 August 1997" |
| 15 | “A resident of Hyderabad with a fever of (≥37·5oC) for at least 3 days from Nov 30, 2016, onwards, without any other cause identified” | “A resident of Hyderabad district with blood-culture confirmed ceftriaxone-resistant S Typhi infection” |
| 93 | NA | "Clinical picture supported by the laboratory parameters: (i) isolation of S.typhi from blood; (ii) a four fold rise in widal agglutinins; (iii) single 'O' and 'H' titre of 1/160 or more" |
| 56 | **Probable Case:**“Acute illness with ⩾3 days of documented fever (temperature, ⩾38.5°C) and ⩾3 other symptoms (headache, malaise, anorexia, nausea, abdominal discomfort, dry cough, or myalgia) without isolation of *S.* Typhi” | “Isolation of *S.* Typhi from stool specimens or blood samples obtained from a resident of Cincinnati or traveler thereto during the period of 2 July through 10 September 2000” |
| 42 | "Any individual who, between October and November 1999, had fever for 3 or more days and at least 3 of the following features: abdominal pain, diarrhea/, nausea/vomiting, malaise/headache, rose sports, or splenomegaly, and who had no other pathogens identified such as malaria." | "Definite diagnosis was confirmed by a positive blood culture for the organism" |
| 90 | **Probable Case:** "A participant at the supper who had a fever equal or superior to 38C for more than 2 days (with no other cause of fever checked by the practitioner) with onset more that 10 days after the supper, and who had either a negative stool or blood culture to Salmonella typhi or did not have a specimen tested."  Case of gastroenteritis (suspected case): "a participant at the supper who had diarrhea (more than three stools per day for at least 1 day) within 12 days of the supper" | "In addition to the fever, a stool or blood specimen cultured positive for S. typhi" |
| 34 | "Surgically diagnosed intestinal perforation consistent with Salmonella Typhi infection or illness characterized by fever and abdominal pain for >= 1 day and at least 1 of the following signs or symptoms: gastrointestinal disruptions, such as vomiting, diarrhea, or constipation, general body weakness, joint pain, headache, clinical suspicion for intestinal perforation, or failure to respond to antimalarial medications --with onset from January 1 to December 31, 2011 in Bundibugyo residents and from August 1, 2009 to December 31, 2011 in residents of the other districts, including Kasese" | NA |
| 65 | "Persistent fever (≥38 °C for >3 days) accompanied by headache, hypoeosinophilia or a positive Widal test in endemic regions and without obvious upper respiratory or urinary tract infections, trauma, or other diagnosed causes of fever." | “Positive culture from blood or stool” |
| 43 | NA | “Either a patient must have had S. typhi isolated by blood culture or, in lieu of blood culture positivity, the patient must have had (1) body temperature ≥38.5°C (measured orally) with a duration of reported fever >= 24 hrs; and (2) >=1 of the following signs and symptoms: fatigue, headache, general malaise, anorexia, abdominal pain or constipation; and a positive Widal test result” (titers given) |

**Supplemental Table 3. Number of Typhoid Outbreaks by Mode of Transmission and Certainty of Source Identified (*n*= 43)**

|  | Suspected Source (n=10)  *[Ref]* | Probable Source (n=8)  *[Ref]* | Confirmed Source (n=25)  *[Ref]* |
| --- | --- | --- | --- |
| Waterborne | 4 | 2 | 18 |
|  | Contamination of drinking water supply: boreholes, municipal water (4) *^52, 65, 83, 85^* | Contamination of drinking water supply [boreholes] (2)*^46, 59^* | Contamination of drinking water supply: municipal water, well, river, spring, reverse osmosis plant, water used to make juice (17). *^15, 34, 38, 41, 42, 47, 49, 51, 57, 60-62, 64, 84, 86, 94, 95^* Recreational water *^58^* |
| Foodborne | 5 | 6 | 6 |
|  | Cake with icing ^87^, Unknown (2) *^43, 63^*, Food handler (2) *^45, 53^* | Orange juice *^80^*, Food handler (3) *^39, 89, 91^* Chicken and rice *^90^* Raw fish *^96^* | Frozen fruit (2) *^66, 67^*, Raw pork/chicken *^39^*, Food handler (3) *^40, 62, 92^* |
| Direct contact | 1 | 0 | 1 |
|  | Psychiatric ward *^48^* |  | Sexual transmission *^56^* |
